# The Association Between Healthy Work Environment and Work Engagement Among Intensive Care Unit Nurses: The Mediating Roles of Self‐Efficacy and Compassion Fatigue

**DOI:** 10.1155/jonm/5655856

**Published:** 2026-09-07

**Authors:** Yi Ming Lu, Shu Wei Zhang, Ming Yue Jia, Xiu Die Liu, Yan Zhang, Guo Zeng Zhang

**Affiliations:** ^1^ Nursing Department, Huaihe Hospital of Henan University, Kaifeng, Henan, China, henu.edu.cn; ^2^ Institute of Nursing and Health, School of Nursing and Health, Henan University, Kaifeng, Henan, China, henu.edu.cn

**Keywords:** compassion fatigue, healthy work environment, intensive care unit nurses, self-efficacy, work engagement

## Abstract

**Background:**

Nurse work engagement is a persistent issue in healthcare. Low engagement reduces professional dedication and sense of belonging, increases the likelihood of turnover, impedes collaboration and treatment continuity, and, ultimately, has a negative impact on care quality and patient safety. The issue warrants particular attention in the demanding context of intensive care. Therefore, maintaining higher levels of work engagement among ICU nurses is important for workforce retention and high‐quality patient care.

**Objective:**

To assess the association between a healthy work environment and work engagement among ICU nurses and to test the hypothesized mediating roles of self‐efficacy and compassion fatigue.

**Methods:**

A cross‐sectional survey involving ICU nurses was conducted at four general hospitals in China from November 2025 to January 2026. The Healthy Work Environment Assessment Tool (HWEAT), Utrecht Work Engagement Scale (UWES), General Self‐Efficacy Scale (GSES), and Compassion Fatigue Short Scale (CF‐Short Scale) were used. Statistical analyses were conducted in SPSS 25.0 and structural equation modeling in AMOS 26.0.

**Results:**

Significant indirect associations were identified through self‐efficacy and compassion fatigue, which together accounted for part of the association between a healthy work environment and work engagement. The results indicated that a healthy work environment was positively associated with self‐efficacy (*β* = 0.67, *p* < 0.001) and work engagement (*β* = 0.46, *p* < 0.001) and negatively associated with compassion fatigue (*β* = −0.51, *p* < 0.001). Additionally, self‐efficacy was positively associated with work engagement (*β* = 0.33, *p* < 0.001) and negatively associated with compassion fatigue (*β* = −0.36, *p* < 0.001). Compassion fatigue was negatively associated with work engagement (*β* = −0.22, *p* < 0.001). The hypothesized model demonstrated acceptable fit: *χ* ^2^/*d* *f* = 3.240, CFI = 0.983, RMSEA = 0.079, and GFI = 0.933.

**Conclusion:**

This study assessed the association between a healthy work environment and work engagement among ICU nurses and examined the indirect roles of self‐efficacy and compassion fatigue. Hospital administrators may consider prioritizing healthy work environments and implementing initiatives that support nurses’ self‐efficacy and address compassion fatigue, given their associations with work engagement. Notably, the cross‐sectional nature of the data precludes causal interpretation; the findings should, therefore, be interpreted as associations rather than causal effects.

**Reporting Method:**

The study was reported in accordance with the Strengthening the Reporting of Observational Studies in Epidemiology (STROBE) guidelines for observational research.

**Implication for Nursing Management:**

The findings indicate that a healthy work environment, self‐efficacy, compassion fatigue, and work engagement are closely interrelated among ICU nurses. Nursing managers may adopt an integrated strategy that combines environmental support with efforts to promote self‐efficacy and address compassion fatigue, thereby potentially supporting work engagement among ICU nurses. Meanwhile, greater attention to the healthy work environment and nurses’ self‐efficacy may help support workforce resilience, care quality, and staff retention.

## 1. Introduction

In recent years, changes in healthcare models and the continued rise in quality standards for nursing services have occurred alongside increasing work pressure and professional challenges among clinical nurses [1]. Therefore, enhancing nurses’ work engagement has become a critical issue in nursing management. Work engagement is a positive occupational state reflected in vigor, dedication, and absorption [2]. Vigor reflects sustained energy, psychological resilience, willingness to expend effort, and persistence when difficulties arise; dedication reflects the perception of meaning, enthusiasm, inspiration, pride, and challenge in one’s work; absorption entails being fully immersed in work tasks and deriving a sense of enjoyment from this engagement [3]. Work engagement is an established marker of occupational well‐being and has been linked to staff retention [3, 4], organizational performance [5, 6], financial outcomes [6], patient safety, mortality, and quality of care [7], as well as improved healthcare quality outcomes [8]. Consistent with findings from neonatal intensive care unit (ICU) nurses [9], work environment quality is robustly associated with professional well‐being outcomes, including compassion fatigue and work engagement [10]. Levels of work engagement vary substantially among nurses worldwide. For instance, studies conducted in China have indicated that clinical nurses often demonstrate moderate levels of work engagement, which are frequently correlated with heightened work‐related stress and greater turnover intention [11]. By comparison, nurses employed in Spanish public hospitals reported greater work engagement, which was positively related to job satisfaction [12]. This difference may reflect the combined influence of individual characteristics and the occupational environment on work engagement.

The demanding nature of nursing care and the severity of patients’ conditions make the performance of ICU nurses especially important [13]. The ICU, as a specialized department within the hospital, is characterized by a high‐intensity work environment and significant psychological demands, requiring nurses to frequently manage critically ill patients and complex resuscitation procedures [14]. In this setting, physicians are primarily responsible for medical decision‐making, whereas nurses have an important role in coordinating care delivery, facilitating efficient care processes, and offering both professional and emotional assistance [15]. ICU nurses typically undergo more structured clinical preparation than their counterparts in general wards and are required to demonstrate greater clinical vigilance and independent practice [15]. However, long working hours, irregular shifts, and substantial professional demands may intensify both occupational and psychological strain, making sustained performance more difficult [16]. Sustained work pressure may gradually deplete nurses’ emotional resources, reduce their focus and professional enthusiasm, and ultimately negatively affect their overall work engagement [17]. Similar patterns of emotional resource depletion have been documented among ICU nurses, where high stress levels were associated with reduced resilience [18]. This suggests that sustained environmental pressure arising from pandemics or chronic under‐resourcing may undermine nurses’ psychological capacity for engagement. Furthermore, due to the stringent professional requirements, high training costs, and difficulty in replacing ICU nurses, their relatively high turnover rate imposes an additional operational burden on hospitals [19]. Improving work engagement may help alleviate this burden. Although healthy work environments are known to support nurse well‐being [20], the psychological pathways linking a healthy work environment to greater self‐efficacy and lower compassion fatigue remain less understood. This study, therefore, examines the direct and indirect associations between a healthy work environment and work engagement.

## 2. Background

### 2.1. A Healthy Work Environment and Work Engagement

A healthy work environment is essential for supporting nurses’ professional well‐being and the delivery of optimal patient care. The World Health Organization describes a healthy work environment as one in which employees and managers jointly pursue ongoing improvements in workplace health, safety, and well‐being while supporting sustainable organizational performance [21]. In intensive care settings, where work is both demanding and complex, a healthy work environment is closely associated with the standard of care provided for critically ill patients [22]. Research indicates that a healthy work environment is associated with nurses’ intention to stay, job satisfaction, clinical decision‐making ability, and quality of team communication [20]. More importantly, a healthy work environment is closely related to nurses’ work engagement [23, 24]. Evidence from a sample of 747 nurses showed that work environment quality was related to work engagement [23]. However, despite these preliminary findings, research specifically examining the association between a healthy work environment and work engagement among ICU nurses remains relatively limited.

**Hypothesis 1.**

A healthy work environment is positively correlated with work engagement.

### 2.2. The Mediating Role of Self‐Efficacy

Self‐efficacy reflects a person’s perceived capacity to carry out a particular action or accomplish a given task successfully [25]. Bandura’s framework identifies mastery and failure experiences, observed performance, emotional responses, and physiological activation as important sources of self‐efficacy [26]. These findings suggest that self‐efficacy is modifiable and may improve through mastery experiences, guided learning, and supportive feedback [27]. In clinical practice, greater self‐efficacy is crucial for nurses, as it enables them to effectively mobilize their knowledge and skills when facing complex medical conditions and high‐pressure situations, thereby demonstrating stronger clinical adaptability and resilience [28]. Previous research has linked a healthy and supportive work environment to stronger self‐efficacy among nurses, partly because such settings may offer more opportunities for positive feedback [25]. Self‐efficacy was also positively associated with nurses’ work engagement [29]. Aviles Gonzalez et al. [30] reported a significant positive association between self‐efficacy and work engagement. Nurses with high self‐efficacy tend to feel more capable of dealing with work challenges, which may encourage more proactive engagement and higher levels of vigor, dedication, and absorption [29].

**Hypothesis 2.**

Self‐efficacy mediates the relationship between a healthy work environment and work engagement.

### 2.3. The Mediating Role of Compassion Fatigue

The concept of compassion fatigue was introduced by Joinson [31] to characterize the exhaustion and functional impairment arising among healthcare professionals under sustained work‐related stress [32]. Coetzee and Klopper [33] characterized compassion fatigue as a progressively accumulating condition associated with the quality of interactions with patients, individual psychological resources, and the level of occupational stress exposure. Compassion fatigue can compromise nurses’ physical and psychological well‐being, diminish care quality, and undermine professional stability [34, 35]. Evidence suggests that the quality of the work environment is related to the emergence and progression of compassion fatigue [36]. Adequate resources, effective management, and organizational support may offer nurses emotional protection and be associated with lower burnout and compassion fatigue [37, 38]. Conversely, poorer workplace conditions, including inadequate support, resource constraints, and ineffective management, have been linked to greater compassion fatigue among nurses [39]. Furthermore, higher compassion fatigue was linked to lower work engagement among nurses [40]. Nurses experiencing compassion fatigue have severely depleted emotional resources, manifesting as decreased work vitality, diminished professional enthusiasm, and difficulty maintaining concentration [41]. This depletion of intrinsic motivation not only increases the risk of clinical errors and compromises patient safety but also directly impairs their ability to remain positive at work [32].

**Hypothesis 3.**

Compassion fatigue serves as a mediator of the association between a healthy work environment and work engagement.

### 2.4. Self‐Efficacy and Compassion Fatigue

From a social cognitive perspective, self‐efficacy represents an important individual psychological resource that can influence how individuals appraise and cope with stressful events, thereby shaping stress‐related outcomes, including compassion fatigue [42]. Among nursing students, greater self‐efficacy was associated with higher compassion satisfaction and lower compassion fatigue [43]. Greater self‐efficacy may enable individuals to manage stress with more confidence and to use coping strategies more actively and effectively, thereby reducing emotional exhaustion and compassion fatigue [44]. In contrast, a healthy work environment may help foster nurses’ self‐efficacy, which may in turn support greater persistence and resilience in work engagement [45]. On the other hand, prolonged compassion fatigue may continuously deplete an individual’s psychological resources, potentially weakening self‐efficacy, increasing vulnerability to work stress, and ultimately impairing work engagement.

**Hypothesis 4.**

Self‐efficacy and compassion fatigue have chain‐mediating roles in the association between a healthy work environment and work engagement.

**FIGURE 1:**
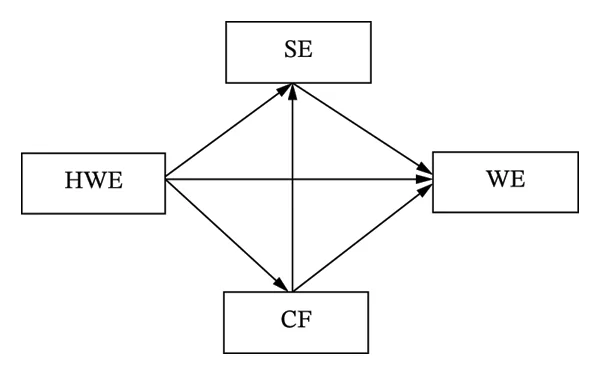
Conceptual framework and hypotheses. *Note*. HWE, healthy work environment; WE, work engagement; SE, self‐efficacy; CF, compassion fatigue.

This study aims to (1) examine the direct association between a healthy work environment and work engagement among ICU nurses; (2) test the mediating role of self‐efficacy in this relationship; (3) test the mediating role of compassion fatigue; and (4) evaluate the chain‐mediating pathway (healthy work environment ⟶ self‐efficacy ⟶ compassion fatigue ⟶ work engagement). In accordance with the study aims, the conceptual framework is presented in Figure 1.

## 3. Methods

### 3.1. Study Design and Participants

ICU nurses were recruited by convenience sampling from four hospitals in Henan Province, China, between November 2025 and January 2026. Eligibility criteria were as follows: (1) holding a valid nursing practice certificate; (2) having worked in an ICU for at least 1 year; and (3) providing informed consent and agreeing to participate voluntarily. At least 1 year of ICU experience was considered necessary to provide participants with adequate exposure to the work environment and sufficient time to develop stable perceptions of work engagement, self‐efficacy, and compassion fatigue. The exclusion criteria were as follows: (1) having chronic or mental‐related diseases and (2) being on personal or medical leave during the data collection period. Sample size adequacy for structural equation modeling was assessed using the criterion of 10 participants for each estimated parameter [46]. As the model contained 31 estimated parameters, the minimum sample size was 310. The recruitment target was set at 400 to allow for potentially incomplete questionnaires.

Before data collection, the research team obtained permission and administrative support from the relevant hospital departments and ICU managers. Questionnaires were distributed to eligible nurses within their respective ICU departments. Completed questionnaires were retrieved within the specified collection period and screened for completeness and response quality before data entry.

### 3.2. Measurement

#### 3.2.1. Participant Characteristics

Information was obtained on gender, age, marital status, highest level of education, employment type, professional title, years of ICU nursing experience, number of night shifts per month, hospital level, and average monthly income in RMB.

#### 3.2.2. Healthy Work Environment

The healthy work environment was assessed using the Healthy Work Environment Assessment Tool, which was developed by the American Association of Critical‐Care Nurses for use in ICU settings [47, 48]. The 18‐item instrument covers six domains: effective decision‐making, appropriate staffing, authentic collaboration, meaningful recognition, skilled communication, and authentic leadership [48]. Items are scored from 1 to 5; higher totals indicate a healthier work environment. The Cronbach’s *α* for the scale was 0.908 in the original validation study and 0.968 in the current study.

#### 3.2.3. Work Engagement

Work engagement was assessed using the Utrecht Work Engagement Scale [49]. This scale consists of 15 items across three dimensions: vigor (6 items), dedication (4 items), and absorption (5 items). Each item is scored from 1 (*never*) to 7 (*always*), with larger totals indicating greater work engagement. The scale showed a Cronbach’s *α* of 0.90 in the original validation and 0.968 in this study.

#### 3.2.4. Self‐Efficacy Scale

Self‐efficacy was evaluated with the Chinese General Self‐Efficacy Scale, developed by Schwarzer and Born [50, 51]. The instrument comprises 10 items rated from 1 (*completely incorrect*) to 4 (*completely correct*). Possible scores range between 10 and 40, and larger values reflect stronger perceived self‐efficacy. Evidence from earlier studies supports the reliability and validity of the instrument. The Cronbach’s *α* obtained in the current sample was 0.937.

#### 3.2.5. Compassion Fatigue

The Compassion Fatigue Short Scale was used to evaluate compassion fatigue [52]. This instrument comprises 13 items across two dimensions: secondary trauma (5 items) and burnout (8 items). Responses are scored from 1 to 10, producing total scores between 13 and 130; larger values indicate more severe compassion fatigue. Severity categories were based on the average item score obtained by dividing the total by 13. Scores below 4 denoted mild fatigue, values from 4 to 7 indicated moderate fatigue, and scores above 7 represented severe fatigue, based on previously validated cutoffs [52]. The developers reported a Cronbach’s *α* coefficient of 0.90 [52]. In the current sample, Cronbach’s *α* was 0.986. Construct validity was confirmed by confirmatory factor analysis, with all factor loadings above 0.60.

### 3.3. Ethical Considerations

A cross‐sectional survey was conducted without any clinical intervention. It was considered to pose minimal risk to participants. Participants provided written informed consent and could withdraw at any time without penalty. Data were deidentified before analysis. The study was approved by the Ethics Committee of Huaihe Hospital of Henan University (No. 2025144) and conducted in accordance with the Declaration of Helsinki.

### 3.4. Statistical Analysis

Data analysis and model construction were performed using SPSS 25.0 and AMOS 26.0. Descriptive statistics summarized participants’ demographic characteristics and the four study variables. Skewness and kurtosis values were within ±2 for all variables, indicating no significant deviation from normality. Pearson correlations were calculated for the four study variables. Overall model fit was evaluated using the chi‐square statistic relative to the degrees of freedom (*χ* ^2^/*d* *f*), goodness‐of‐fit index (GFI), adjusted goodness‐of‐fit index (AGFI), standardized root mean square residual (SRMR), normed fit index (NFI), relative fit index (RFI), incremental fit index (IFI), Tucker–Lewis index (TLI), comparative fit index (CFI), and root mean square error of approximation (RMSEA). Lower values of *χ* ^2^/*d* *f*, SRMR, and RMSEA indicate better model fit, whereas higher GFI, AGFI, NFI, RFI, IFI, TLI, and CFI values indicate better fit [53]. Indirect effects were evaluated using a bias‐corrected bootstrap procedure with 5000 resamples, and corresponding 95% confidence intervals (CIs) were estimated [54]. All tests were two‐sided, with *p* < 0.05 considered statistically significant.

## 4. Results

### 4.1. The Demographic Characteristics of the Participants

Of the 400 questionnaires distributed, 36 were excluded because they were incomplete or invalid. The remaining 364 questionnaires were included in the final analysis, corresponding to a valid response rate of 91.0%. In this final analytical sample, all study variables had complete data; thus, no missing data imputation was required. Nurses educated to the bachelor’s level constituted the largest subgroup (66.2%). Most participants had 3–5 years of ICU experience (67.6%). The predominance of nurses with 3–5 years of experience suggests that the findings may primarily reflect ICU nurses at relatively early career stages. Participant characteristics are presented in Table 1 (*N* = 364).

**TABLE 1:** Demographic characteristics of the participants (*N* = 364).

Variables		*n*	%
Gender	Men	72	19.8
	Women	292	80.2

Age (years)	≤ 30	152	41.8
	31–40	135	37.1
	≥ 41	77	21.2

Marital status	Single	129	35.4
	Married	224	61.5
	Divorced/widowed	11	3.0

Highest level of education	Junior college or below	89	24.5
	Bachelor’s	241	66.2
	Master’s or above	34	9.3

Employment relationship	Permanent staff	65	17.9
	Contractual staff	265	72.8
	Other	34	9.3

Title	Nurse	248	68.1
	Nurse‐in‐charge	82	22.5
	Deputy chief nurse or above	34	9.3

Years as an ICU nurse (years)	≤ 2	56	15.4
	3–5	246	67.6
	6–10	42	11.5
	> 10	20	5.5

Night shifts per month	0 – 3 times	47	12.9
	4 – 6 times	248	68.1
	≥ 7 times	69	19.0

Hospital level	Tertiary	273	75.0
	Secondary	91	25.0

Average monthly income (RMB)	< 4000	85	23.4
	4000–6000	221	60.7
	6001–8000	58	15.9

### 4.2. Pearson’s Correlation Analysis

The scores for healthy work environment, work engagement, compassion fatigue, and self‐efficacy were 64.85 ± 17.40, 59.07 ± 19.96, 52.05 ± 35.33, and 30.47 ± 9.60, respectively (Table 2). The mean item score for compassion fatigue was approximately 4.00 (52.05/13), indicating that participants, on average, reported moderate levels of compassion fatigue (categorized as 4–7). Pearson correlation analysis showed that all key variables were significantly correlated. A positive correlation was found between a healthy work environment and work engagement (*r* = 0.815, *p* < 0.01). A healthy work environment was negatively correlated with compassion fatigue (*r* = −0.728, *p* < 0.01) and positively correlated with self‐efficacy (*r* = 0.662, *p* < 0.01). Furthermore, work engagement was negatively correlated with compassion fatigue (*r* = −0.741, *p* < 0.01) and positively correlated with self‐efficacy (*r* = 0.782, *p* < 0.01). Compassion fatigue and self‐efficacy were also significantly negatively correlated (*r* = −0.685, *p* < 0.01). Although a healthy work environment and work engagement were relatively highly correlated (*r* = 0.815), the correlation remained below the commonly used threshold of 0.85. Variance inflation factors ranged from 2.1 to 2.5, indicating no problematic multicollinearity.

**TABLE 2:** Descriptive statistics and correlation analysis (*N* = 364).

	HWE	WE	CF	SE	Mean	Standard deviation
HWE	1				64.85	17.40
WE	0.815^∗∗^	1			59.07	19.96
CF	−0.728^∗∗^	−0.741^∗∗^	1		52.05	35.33
SE	0.662^∗∗^	0.782^∗∗^	−0.685^∗∗^	1	30.47	9.60

Abbreviations: CF, compassion fatigue; HWE, healthy work environment; SE, self‐efficacy; WE, work engagement.

^∗∗^ *p* < 0.01.

### 4.3. Mediating Effect Analysis and Hypothesis Path Testing

**FIGURE 2:**
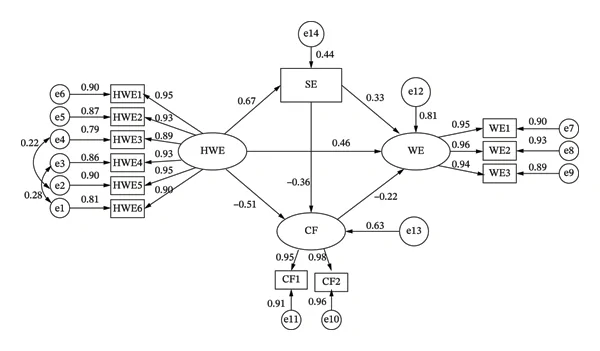
Standardized structural paths among the study variables. *Note*. HWE, healthy work environment; WE, work engagement; SE, self‐efficacy; CF, compassion fatigue.

Structural equation modeling was performed in AMOS to assess the mediating pathways [55], and the findings are presented in Figure 2 and Table 3. Path analysis identified positive associations between a healthy work environment and both self‐efficacy (*β* = 0.67, *p* < 0.001) and work engagement (*β* = 0.46, *p* < 0.001), together with an inverse association with compassion fatigue (*β* = −0.51, *p* < 0.001). Furthermore, self‐efficacy was significantly and positively associated with work engagement (*β* = 0.33, *p* < 0.001) and significantly and negatively associated with compassion fatigue (*β* = −0.36, *p* < 0.001). Compassion fatigue was also inversely associated with work engagement (*β* = −0.22, *p* < 0.001). Standardized path coefficients (*β*) are reported unless otherwise specified. Unstandardized coefficients (B) are presented in Table 3.

**TABLE 3:** Total, direct, and indirect associations linking a healthy work environment to work engagement.

Effect	Path relationship	Effect	Bootstrap SE	Bootstrap 95% CI	*p* value
Direct effect	HWE ⟶ WE	1.488	0.160	1.185, 1.818	< 0.001

Indirect effect	HWE ⟶ SE ⟶ WE	0.697	0.082	0.535, 0.860	< 0.001
	HWE ⟶ CF ⟶ WE	0.217	0.084	0.045, 0.382	0.013
	HWE ⟶ SE ⟶ CF ⟶ WE	0.101	0.047	0.018, 0.202	0.013

Total indirect effect		1.015	0.125	0.754, 1.246	< 0.001

Total effect	HWE ⟶ WE	2.503	0.093	2.319, 2.687	< 0.001

Abbreviations: CF, compassion fatigue; HWE, healthy work environment; SE, self‐efficacy; WE, work engagement.

Subsequently, the mediating roles of self‐efficacy and compassion fatigue in the association of a healthy work environment with work engagement were examined using a bootstrapping approach with bias‐corrected CIs. Based on 5000 bootstrap resamples, the analysis revealed a significant total indirect effect of 1.015 (Bootstrap 95% CI: 0.754, 1.246, *p* < 0.001). The total indirect effect was 1.015, representing 40.6% of the total effect of 2.503 and supporting partial mediation. Specifically, three significant indirect pathways contributed to this effect.

The first pathway represented the indirect influence of a healthy work environment on work engagement through self‐efficacy, that is, healthy work environment ⟶ self‐efficacy ⟶ work engagement. The indirect effect was 0.697, bootstrap 95% CI: (0.535, 0.860), and *p* < 0.001.

The second pathway represented the indirect association between a healthy work environment and work engagement through compassion fatigue, that is, healthy work environment ⟶ compassion fatigue ⟶ work engagement. The indirect effect was 0.217, bootstrap 95% CI: (0.045, 0.382), and *p* = 0.013.

The third indirect pathway involved self‐efficacy and compassion fatigue, that is, healthy work environment ⟶ self‐efficacy ⟶ compassion fatigue ⟶ work engagement. The indirect effect was 0.101, bootstrap 95% CI: (0.018, 0.202), and *p* = 0.013.

### 4.4. Model Fit Analysis

The hypothesized model included healthy work environment, work engagement, self‐efficacy, and compassion fatigue. The model fit indices are as follows: *χ* ^2^/*d* *f* = 3.240 (< 5.0), GFI = 0.933 (> 0.90), CFI = 0.983 (> 0.90), AGFI = 0.892 (close to 0.90), TLI = 0.976 (> 0.90), IFI = 0.983 (> 0.90), and RMSEA = 0.079 (< 0.08). The AGFI value of 0.892 is marginally below the 0.90 threshold but is considered acceptable given the complexity of the model and the sample size [56]. The SRMR was 0.021, indicating acceptable fit [53]. Overall, the fit indices supported an acceptable model fit (Table 4).

**TABLE 4:** Model fit summary.

Model fit	*χ* ^2^/*d* *f*	GFI	AGFI	SRMR	NFI	RFI	IFI	TLI	CFI	RMSEA
	3.240	0.933	0.892	0.021	0.975	0.966	0.983	0.976	0.983	0.079

## 5. Discussion

### 5.1. Summary of Principal Findings

The principal findings of this study were as follows: (1) A healthy work environment was positively associated with work engagement among ICU nurses; (2) self‐efficacy and compassion fatigue each showed significant indirect effects; and (3) a significant chain‐mediation pathway was identified (healthy work environment ⟶ self‐efficacy ⟶ compassion fatigue ⟶ work engagement). In addition, although our cross‐sectional design precludes causal inference, it is plausible that nurses with higher work engagement perceive their work environment more positively (reverse causality). Longitudinal studies are needed to establish temporal ordering.

### 5.2. Comparison With Existing Literature

In this sample, ICU nurses showed moderate work engagement, consistent with most previous studies [57, 58], but slightly lower than that reported in a study from Egypt [59]. This moderate level highlights the need to further understand factors associated with work engagement in ICU nurses. In this regard, a healthy work environment was positively and significantly associated with work engagement, providing support for Hypothesis 1. Previous studies have shown that lower work engagement and greater turnover intention among nurses are associated with adverse workplace conditions, restricted career progression, inadequate leadership support, ethical and occupational stress, disconnects between education and practice, and workplace bullying [60–62]. The positive association between a healthy work environment and work engagement (*β* = 0.46) is broadly consistent with findings among neonatal ICU nurses, where the work environment was also associated with professional quality‐of‐life outcomes [9].

The strong association between a healthy work environment and work engagement should be interpreted with caution. Although closely related, the two constructs are conceptually distinct: a healthy work environment reflects nurses’ perceptions of organizational conditions, such as leadership, communication, collaboration, staffing adequacy, decision involvement, and access to professional resources [48], whereas work engagement describes nurses’ positive work‐related state, manifested in vigor, dedication, and absorption [2]. Their close association may be particularly salient in the ICU context, but its magnitude may also partly reflect the concurrent use of self‐report measures and reciprocal perceptions. Therefore, the observed correlation should be interpreted cautiously as indicating a close association between the two constructs.

Moreover, because the present study was cross‐sectional, alternative directional interpretations are also plausible. For example, nurses with higher work engagement may perceive their work environment more positively, suggesting that the observed association may be reciprocal rather than unidirectional. These two directional interpretations are not mutually exclusive and may coexist. From a practical perspective, establishing and maintaining a healthy work environment may require support at multiple levels. First, ongoing and open communication and interaction between staff and leaders are essential for cultivating a healthy work environment [57], helping to strengthen shared responsibility and promote effective teamwork [63, 64]. Second, mutual respect in the nursing workplace is associated with the development and maintenance of a healthy environment [65]. Therefore, collaborative efforts by nursing leaders and clinical nurses to promote a healthy work environment may help support nurses’ work engagement. These findings may have implications beyond work engagement, as improving leadership, staffing adequacy and organizational support has also been associated with a lower risk of workplace violence against nurses, further emphasizing the strategic value of investing in healthy work environments [66].

The results supported Hypothesis 2, with self‐efficacy showing a significant indirect association between a healthy work environment and work engagement. This finding aligns with prior research [67], suggesting that supportive and healthy work environments are associated with greater self‐efficacy among nurses and, in turn, more active engagement in clinical work. Factors such as effective physician–nurse collaboration, a respectful team atmosphere, and adequate resource support have all been shown to be positively associated with nurses’ self‐efficacy and are linked to greater confidence and proactive adaptation when facing work challenges [68, 69]. Self‐efficacy was positively associated with work engagement. Nurses with greater self‐efficacy may show more sustained work engagement, together with stronger psychological resilience and problem‐solving capacity when difficulties arise [70]. This intrinsic motivation enables nurses to remain vigorous, dedicated, and fully absorbed in their work [45].

Evidence from other high‐stress nursing contexts similarly supports the indirect role of self‐efficacy between a healthy work environment and work engagement. Comparable evidence has been reported among Palestinian ICU nurses, where work environment quality was related to professional quality of life [9]. In addition, resilience—a construct conceptually related to self‐efficacy—was identified as an intermediary mechanism linking stress to psychological outcomes [18, 71]. Efforts to strengthen nurses’ self‐efficacy could involve continuing professional education and training, adequate access to protective equipment, policy‐level protection of income and welfare benefits, more manageable workloads and nurse‐to‐patient ratios, and the provision of psychological support services [72]. Such measures may help nurses manage professional challenges, maintain self‐efficacy, and support work engagement.

### 5.3. The Mediating Role of Compassion Fatigue

The results supported Hypothesis 3, with compassion fatigue showing a significant indirect association between a healthy work environment and work engagement. This finding aligns with Aqtam et al. [18], who linked greater stress among ICU nurses to lower resilience, a pattern comparable to the association of an unsupportive work environment with compassion fatigue and reduced work engagement. The findings indicate a significant negative correlation between a healthy work environment and compassion fatigue, with a healthier work environment being statistically associated with lower levels of compassion fatigue. Similar results have been reported in earlier studies [73, 74]. Previous evidence indicates that occupational stressors, including inadequate staffing and limited resources, are associated with greater compassion fatigue [75]. In contrast, a well‐supported and effectively managed work environment is associated with stronger professional motivation and lower levels of burnout and compassion fatigue [37, 76].

Compassion fatigue has been associated with emotional distancing, reduced professional engagement, and poorer occupational well‐being [75, 77–80]. Accordingly, higher compassion fatigue may coincide with weaker professional identity, reduced emotional connection, and lower work engagement. To address compassion fatigue and support nurses’ work engagement, coordinated multilevel strategies may be considered. At the hospital level, organizational environments and resource allocation should be optimized and routine psychological support mechanisms should be established. At the departmental level, a collaborative and trusting team atmosphere should be fostered, flexible scheduling should be implemented, and team building should be strengthened. Individual‐focused measures may include emotion regulation training and support for self‐efficacy, with the aim of improving nurses’ capacity to manage occupational stress.

### 5.4. The Chain‐Mediation Pathway

The results supported Hypothesis 4 by identifying a significant chain‐mediated association linking a healthy work environment, self‐efficacy, compassion fatigue, and work engagement. The chain‐mediated indirect effect (0.101) was smaller than the indirect effect through self‐efficacy alone (0.697) and through compassion fatigue alone (0.217). This suggests that self‐efficacy may account for a larger proportion of the indirect association in our sample, while compassion fatigue may represent a secondary pathway. The present study extends previous research by examining self‐efficacy and compassion fatigue within a single chain‐mediation model among ICU nurses. Rather than considering these psychological factors separately, the findings suggest that they may represent interrelated components of the association between a healthy work environment and work engagement, thereby providing a more integrated understanding of this relationship.

Specifically, a healthy work environment was statistically associated with greater self‐efficacy among nurses [81], while greater self‐efficacy was linked to lower compassion fatigue and, in turn, greater work engagement. Conversely, an inadequately supportive work environment may be associated with lower self‐efficacy, which may leave nurses more susceptible to compassion fatigue and, in turn, lower work engagement [82]. Similar patterns of moral distress and resilience erosion have been documented among nurses in West Bank governmental hospitals [83], suggesting that institutional support, whether absent due to conflict or chronic under‐resourcing, may produce comparable psychological consequences across settings. Beyond organizational factors, future longitudinal and multilevel studies should examine whether patient‐level nursing complexity modifies the relationships among healthy work environment, self‐efficacy, compassion fatigue, and work engagement, as higher complexity has been associated with clinical deterioration, ICU transfer, and other adverse outcomes [84–87]. Therefore, fostering a healthy work environment may help support nurses’ work engagement and the delivery of high‐quality care to critically ill patients.

### 5.5. Implications for Nursing Management

For practice: nursing managers may consider implementing routine self‐efficacy assessments and providing targeted mastery experiences (e.g., simulation training and constructive feedback) to strengthen this psychological resource. For policy: hospital administrators may consider reviewing staffing adequacy and workload allocation, while also strengthening access to psychological support services, as these organizational factors may be associated with lower compassion fatigue. Furthermore, the chain‐mediation findings suggest that nurses’ work engagement may involve interrelationships among organizational conditions, psychological resources, and emotional burden. Nursing managers may, therefore, consider understanding and supporting nurses’ work engagement from both organizational and individual psychological perspectives.

## 6. Limitations

First, the cross‐sectional design does not support causal inference. Although theoretically grounded, reverse causality (e.g., engaged nurses perceiving their environment as healthier) cannot be ruled out. Second, the use of self‐report measures may have increased susceptibility to common method variance and socially desirable responding. Third, convenience sampling from four hospitals in Henan Province, China, limits generalizability to other regions, hospital types, or cultural contexts. Fourth, the predominance of nurses with 3–5 years of experience (67.6%) means that the findings may not represent late‐career ICU nurses. Fifth, healthy work environment and work engagement were strongly correlated in this sample. Although the two constructs are conceptually distinct, the observed association may partly reflect reliance on self‐reported data and the potential for reciprocal perceptions. Therefore, future studies using longitudinal, multisource, or latent‐variable approaches are needed to further examine their empirical distinctiveness. Sixth, the analysis did not account for several potential confounders, including individual characteristics such as resilience and trait anxiety, as well as organizational factors such as patterns of team collaboration and workplace culture. Finally, temporal changes in self‐efficacy and compassion fatigue, together with their associations with the healthy work environment, could not be evaluated because of the cross‐sectional study design.

## 7. Conclusion

This study demonstrated meaningful relationships among a healthy work environment, self‐efficacy, compassion fatigue, and work engagement among ICU nurses. More specifically, a healthy work environment was linked to greater work engagement among ICU nurses, with self‐efficacy and compassion fatigue involved in the indirect associations between the two. Future studies may consider longitudinal, interventional, and cross‐cultural approaches to examine temporal relationships, explore the generalizability of the findings across healthcare settings, and determine whether interventions targeting self‐efficacy are followed by changes in compassion fatigue and work engagement over time.

## Author Contributions

Yi Ming Lu: conceptualization, methodology, investigation, data curation, formal analysis, validation, visualization, writing–original draft, and writing–review and editing.

Shu Wei Zhang: methodology, investigation, data curation, formal analysis, and writing–review and editing.

Ming Yue Jia: investigation, data curation, validation, formal analysis, and writing–review and editing.

Xiu Die Liu: investigation, resources, data curation, and writing–review and editing.

Yan Zhang: conceptualization, resources, investigation, project administration, supervision, and writing–review and editing.

Guo Zeng Zhang: conceptualization, methodology, supervision, project administration, visualization, and writing–review and editing.

## Funding

No specific funding was received for this study.

## Disclosure

The authors report no disclosures.

## Ethics Statement

The study received approval from the Ethics Committee of Huaihe Hospital of Henan University (Approval No. 2025144) and adhered to the principles of the Declaration of Helsinki.

## Conflicts of Interest

The authors declare no conflicts of interest.

## Supporting Information

Additional supporting information can be found online in the Supporting Information section.

## Supporting information


**Supporting Information** STROBE_checklist_cross‐sectional.

## Data Availability

The data that support the findings of this study are available on request from the corresponding authors. The data are not publicly available due to privacy or ethical restrictions.
